# Survival estimates across five life stages of redfin (*Perca fluviatilis*) exposed to simulated pumped-storage hydropower stressors

**DOI:** 10.1093/conphys/coac017

**Published:** 2022-04-20

**Authors:** Katherine E Doyle, Nathan Ning, Luiz G M Silva, Eduardo M Brambilla, Z Daniel Deng, Tao Fu, Craig Boys, Wayne Robinson, Jan A du Preez, Lee J Baumgartner

**Affiliations:** Institute for Land, Water and Society, Charles Sturt University, Elizabeth Mitchell Drive, Thurgoona New South Wales 2640, Australia; Institute for Land, Water and Society, Charles Sturt University, Elizabeth Mitchell Drive, Thurgoona New South Wales 2640, Australia; Institute for Land, Water and Society, Charles Sturt University, Elizabeth Mitchell Drive, Thurgoona New South Wales 2640, Australia; Stocker Lab, Institute of Environmental Sciences, Department of Civil, Environmental and Geomatics Engineering, ETH-Zurich, 8093 Zurich, Switzerland; Departamento de Zoologia, Instituto de Biociências, UNESP–Universidade Estadual Paulista, Caixa Postal 510, 18618-970 Botucatu, São Paulo, Brazil; Pacific Northwest National Laboratory, Hydrology Group, 902 Battelle Blvd, Richland, WA 99352,USA; Pacific Northwest National Laboratory, Hydrology Group, 902 Battelle Blvd, Richland, WA 99352,USA; Institute for Land, Water and Society, Charles Sturt University, Elizabeth Mitchell Drive, Thurgoona New South Wales 2640, Australia; New South Wales Department of Primary Industries, Port Stephens Fisheries Institute, Taylors Beach Road, Taylors Beach NSW 2316, Australia; Institute for Land, Water and Society, Charles Sturt University, Elizabeth Mitchell Drive, Thurgoona New South Wales 2640, Australia; JAD Systems LLC, 3023 Creek Manor Drive, Kingswood, TX 77339, USA; Institute for Land, Water and Society, Charles Sturt University, Elizabeth Mitchell Drive, Thurgoona New South Wales 2640, Australia

**Keywords:** shear, pressurization, invasive species, fish passage, European perch, Blade strike

## Abstract

The global prevalence of pumped-storage hydropower (PSH) is expected to grow exponentially as countries transition to renewable energy sources. Compared to conventional hydropower, little is currently known regarding PSH impacts on aquatic biota. This study estimated the survival of five life stages (egg, two larval stages, juvenile and adult) of redfin (European) perch (*Perca fluviatilis*) following passage through a PSH facility during the pumping phase. This was achieved by simulating the individual stressors expected to occur during passage through a 2000-MW PSH facility using laboratory-simulated (shear strain and extreme compression) and modelling (blade strike, BS) approaches. Our results indicate that redfin could survive the shear, pressure and BS stressors expected within the PSH facility, but impacts varied among life stages. Juvenile survival was >70% across all shear strain rates, while the survival of eggs and larvae declined markedly as strain rate increased. All life stages had high survival when exposed to rapid compression and BS. The high survival of redfin to the stressors tested suggests the PSH facility could facilitate the passage of redfin during the pumping phase from the lower to the higher elevation reservoir. This outcome would be welcomed in situations where the species is native, but could have adverse implications for the conservation of native biota where the species is considered a pest.

## Teaser

Pumped-storage hydropower facilities may facilitate the transfer of redfin (*Perca fluviatilis*) from lower to higher elevation reservoirs and/or among catchments. This would be welcomed in situations where the species is native, but could have detrimental implications for the conservation of native biota where the species is considered a pest.

## Introduction

Global efforts to transition from fossil fuel-based electricity systems to an energy matrix that includes more renewable technologies (e.g. wind, solar, hydropower) have gained substantial traction in recent decades (Beaudin *et al.,* 2010). Bulk electrical energy storage, of which over 99% is composed of pumped-storage hydropower (PSH), can help overcome the problems of intermittency of wind and solar technologies and improve grid stability (Rehman *et al.,* 2015). Subsequently, there has been an enormous amount of interest in PSH, with global models identifying between 616 000 (Stocks *et al.,* 2020) and 5.1 million potential developments (Hunt *et al.,* 2020), and many of these already operational or under development (International Hydropower Association, 2020).

Investment in PSH of this scale is not without environmental risk. It is well established that conventional hydropower facilities contribute to the injury and mortality of fish (Čada, 2001), even leading to fisheries declines (Trumbo *et al.,* 2014). This occurs when fish become entrained during downstream passage over and through hydropower infrastructure (Algera *et al.,* 2020). Fish entrained in hydropower turbines, in particular, can be exposed to hydraulic stressors such as elevated fluid shear (Čada *et al.,* 2007; Deng *et al.,* 2005) and extreme pressure variations (Brown *et al.,* 2014; Pflugrath *et al.,* 2019) and physical stressors such as turbine blade strike (BS) and mechanical force (Deng *et al.,* 2011; Romero-Gomez and Richmond, 2014). Whether a fish survives passage will depend on several factors, including the route of passage, the severity of the stressor and the vulnerability of the fish species or life stage entrained (Colotelo *et al.,* 2016; Coutant and Whitney, 2000; Mathur *et al.,* 2018).

**Figure 1:**
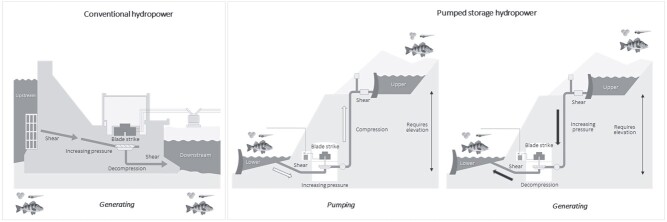
Schematic of a conventional hydropower facility (left) and a PSH facility during pumping (middle) and power generation phases (right), showing the different hydraulic (decompression, compression, shear) and physical (BS) stressors entrained fish, eggs and larvae may be exposed to. Arrows indicate water flow and fish direction through each facility. For the conventional scenario, ‘upstream’ and ‘downstream’ depict the river flow direction. For the PSH scenario, ‘lower’ refers to the lower reservoir and ‘upper’ is the upper reservoir. Diagram not to scale.

In comparison to conventional hydropower, the impact of PSH on aquatic biota is not well documented in the published literature, despite its potential as a promising energy source. However, there is reason to believe that the effects of PSH on fish are different to conventional hydropower based on how they operate. During electricity generation mode, PSH operates in much the same way as conventional hydropower, using the hydrostatic pressure across an operating head to generate power as water flows downstream through the turbines (Rehman *et al.,* 2015). An entrained fish passing through the facility will be exposed to increasing pressure as it approaches the turbine, followed by rapid decompression. It may then be exposed to BS and shear depending on the route of passage as it passes the turbine, with shear continuing on the downstream side of the turbine (Fig. 1). The difference between conventional hydropower and PSH is that the latter has an additional pumping phase, where reversible (usually Francis) turbines are used during off-peak operations to return water from a lower-elevation reservoir to an upper-elevation reservoir (Yang, 2016). These reservoirs are usually disconnected (closed-loop PSH) from naturally connected waterways, although some may be continuously connected to such waterways (open-loop PSH) (Saulsbury, 2020).

During pumping mode, the PSH stressors act somewhat differently to conventional hydropower. Fish will experience increasing pressure as they move towards the turbine, then potentially BS and fluid shear during turbine passage. The fundamental difference between the pumping and generating modes is that when the fish pass through the turbine during the former mode, they experience a rapid (within milliseconds) and extreme (sometimes >7000 kPa) compression rather than a rapid decompression, which is caused by the head differential created from moving water back to the upper reservoir (Fig. 1). Most studies regarding turbine passage focus on the effects of rapid decompression on fish through conventional hydropower turbines (e.g. Boys *et al.,* 2016; Brown *et al.,* 2014; Stephenson *et al.,* 2010). There are only a few earlier studies that examine the impacts of compression on fish through smaller PSH facilities, at pressures less than ~5000 kPa (Foye and Scott, 1965; Lampert, 1976; Rowley, 1955).

The impacts of passage through PSH facilities are likely to affect all life stages of fish, but they may be more significant at specific points in the life history than in others (Brown *et al.,* 2014) and among fish species (Colotelo *et al.,* 2018). For example, conventional hydropower impact studies have shown that shear stress is a significant source of mortality for fish during early life history (Navarro *et al.,* 2019), while BS becomes more significant as fish get larger (Coutant and Whitney, 2000; Deng *et al.,* 2007). Therefore, where there is potential for entrainment or passage of multiple life stages of fish (Suzuki *et al.,* 2011), it is crucial to test the effects of hydraulic and physical stressors across the life stages.

We investigated the effect of simulated PSH passage on the survival of five life stages (egg, two larval stages, juvenile and adult) of redfin (European) perch (*Perca fluviatilis* Linnaeus, 1758, hereafter ‘redfin’) using laboratory-based (shear strain and extreme compression) and modelling (BS) approaches. The three stressors—shear stress, extreme compression and turbine BS—were based on the conditions likely to occur during the pumping phase of a 2000-MW open-loop PSH facility under development in south-eastern Australia. Redfin were chosen because they are widely distributed, occurring in riverine and lake habitats (Froese and Pauly, 2019) where there is a good chance that PSH will be developed. In their native range, they are a valued recreational species, whereas in other areas they are considered a noxious pest species. We then discuss aquatic conservation implications associated with the design- and operational-related factors of PSH. Specifically, we consider how this information can be used by fisheries managers, whether they are trying to protect a valuable fish species, or manage the spread of an unwanted pest species. To our knowledge, this is the first study to investigate multiple hydraulic and physical stressors expected during fish passage at a large PSH and to consider a full range of life stages for a species.

## Material and methods

### Study species

Redfin are native to Eurasia and have been introduced as a sport fish to several southern hemisphere countries including Australia, New Zealand and South Africa (Froese and Pauly, 2019; Hammer and Walker, 2004). They are one of the most widely distributed freshwater fishes in the northern hemisphere where they are valued by recreational anglers (Arlinghaus and Mehner, 2004; Vainikka *et al.,* 2012). In Australia, although they are also valued as an angling species, this value is outweighed by the ecological damage they can cause to native fish populations. Indeed, redfin prey upon and/or compete with some native fish and decapod species (Morgan *et al.,* 2003; Rowe *et al.,* 2007) and can carry the Epizootic Haematopoietic Necrosis Virus—a virus that is potentially lethal to rainbow trout (*Oncorhynchus mykiss*) and threatened Macquarie perch (*Macquaria australasica*) (Langdon, 1989; Whittington and Reddacliff, 1995). Consequently, redfin are listed as a noxious pest species by several fisheries agencies (IFS, 2020; NSW DPI, 2020; PIRSA, 2020), and their unintentional transfer and range expansion by PSH facilities could have grave conservation implications for native aquatic biota (Normyle and Pittock, 2020).

Potential entrainment of any life stages of redfin into a hydropower or PSH intake will depend on the individual features of the intake, primarily depth, velocity and operational frequency. The depth range that juvenile (12–15 m; Vejřík *et al.,* 2016) and larval (13–14 m; Čech *et al.,* 2005) redfin have been observed to occupy in reservoirs overlaps with the intakes of most PSH facilities, as does the depth range observed for redfin spawning (0.2–12 m; Gillet and Dubois, 1995; Smith *et al.,* 2001). Given redfin typically spawn in littoral marginal habitats (Gillet and Dubois, 1995), the entrainment of eggs is less likely but cannot be excluded.

### Fish husbandry

Juvenile and adult redfin were collected from Bethungra Reservoir, New South Wales, Australia (34° 45′ 46.89′′ S, 147° 54′ 14.42″ E), between August and September 2018. Juveniles were caught using boat mounted electrofishing (7.5 GPP Smith-Root) and adults were caught in gill nets (1.8 m drop, 25 m long, 60–80 mm mesh). Fish were transported to the Charles Sturt University Fish Laboratory (Albury, New South Wales) for PSH simulation experiments. Fish were quarantined upon arrival in 1000-l tanks and given a prophylactic salt treatment (5 ppt for 1 week). After quarantine, juveniles and adults were held in separate 1000-l tanks at a density of <2.5 kg/1000 l and salinity was 2.5 ppt throughout the study. Tap water was dechlorinated with Safe™ (1 g/1000 l) and cycled through a biological filter (AST Polygeyser Filter Model 4000) fitted to each tank. Water quality was measured daily (Supplementary Table 1) and maintained with a weekly 25% water change. Juvenile and adult redfin were fed daily on frozen bloodworms, live earthworms and Skretting Ltd floating pellets (3–5 mm).

Fecund adults were identified and moved to a 1000-l brood stock tank at a ratio of 1 female:2 males (Rougeot and Toner, 2008). The timing of redfin housing coincided with their natural reproductive season in the southern hemisphere (September to October). Brood fish naturally spawned on lobster spawning mops when temperatures reached 12°C. Fertilized egg ribbons were transferred into aerated 20-l buckets for embryogenic development until they were used for egg experiments or hatched for larval experiments.

Egg development was monitored daily by assessing samples of each ribbon under a microscope (Olympus SZ2-ILST). Eggs were ready for experimentation once their eyes were visible and embryo movements were initiated (Supplementary Fig. 1; O3 stage described by Alix *et al.,* 2015). Redfin larvae were held in glass aquaria [450 mm (L) × 290 mm (W) × 350 mm (D)] containing a sponge filter (Aqua One Filter 60 Breeder). Larval development was described as the number of days post-hatching (DPH). Following the absorption of their yolk sac (~5 DPH), larvae were fed *Artemia* spp. nauplii five times a day. Larvae were used for experimentation at two phases: 12–18 DPH (O5 stage; Alix *et al.,* 2015; first coordinated movements and oral feeding) and 28–30 DPH (organogenesis completed). For a summary of redfin biometrics for each experiment (shear, pressure, BS) and life stage, see Supplementary Table 2.

### PSH simulations

**Figure 2:**
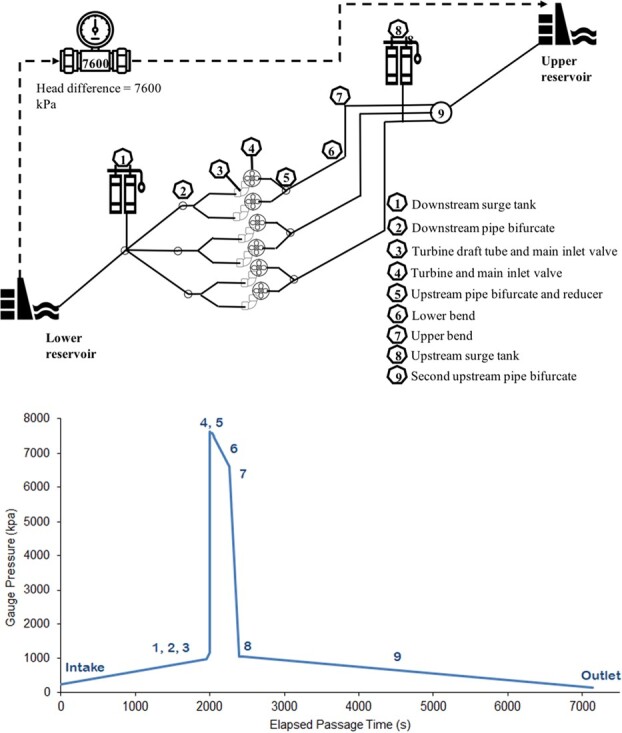
Schematic of the PSH (top) and the expected pressure profile (bottom) generated during full pumping capacity (i.e. six turbines operating). Numbers 1–9 identify different locations in the facility. The head difference reflects the lower reservoir operating at full supply level and upper reservoir at minimum operating level.

The specifications of the stressors under investigation were based on those expected during the pumping phase at a six-Francis turbine 2000-MW open-loop PSH facility (currently under development in Australia). Most PSH facilities are commonly in the range of 1000–1500 MW, with some as large as 2000–3000 MW (Rehman *et al.,* 2015). As the hydraulic and physical stressors tend to scale with the size of the hydropower operation (Neitzel *et al.,* 2004), the present study represents some of the highest stressor situations for fish passing through a PSH facility. The simulations assumed fish were entrained into the PSH at the lower elevation reservoir and pumped to a higher elevation reservoir across a head differential of 800 m (and therefore a maximum pressure of 7600 kPa). These head differential and associated hydrostatic pressure values were slightly higher than those for other large PSH facilities, such as the 1330 MW Ingula scheme in South Africa (head differential of approximately 500 m and an associated maximum hydrostatic pressure of around 5000 kPa; Du Plessis and Twine (2013)), and the 2400 MW Attaqa Mountain scheme being planned for development in Egypt (head differential of approximately 600 m and an associated maximum hydrostatic pressure of around 6000 kPa; Stocks *et al.* (2020)). During a passage event, fish passed a Francis reversible turbine where they could be exposed to BS, then passed through a series of structures before ascending to the upper reservoir (Fig. 2). The profile and passage time simulated for the pressure tests were modelled by the power company and supplied to us. They reflected all six turbines operating, which was expected to be the most extreme scenario for a passing fish (Fig. 2). We tested shear rates ranging from 0 to 18 530 s^−1^, also based on modelling by the power company. These shear rates were higher than those tested in other studies (e.g. Navarro *et al.,* 2019: 626–2002 s^−1^), but were designed to incorporate the range of levels generated by a PSH facility, where fast flowing water passes near internal structures such as draft tubes, wicket gates, spiral casings, runner blade leading edge and stay vanes (Čada, 2001).

### Shear experiments

A shear flume made of a plexiglass tube [1.95 m (L) × 0.44 m (D)] connected to a submerged jet at one end and a reservoir tank [2.10 m (L) × 2.10 m (W) × 0.9 m (D)] at the other was used to generate different shear strain rates. The flume is described in detail in Navarro *et al.* (2019). Different shear strain rates were created by accelerating flow through a conical nozzle in the entry of the plexiglass tube, reducing the flow diameter from 15 to 5 cm over a distance of 26 cm. A pressure gauge ranging from 0 to 45 psi (resolution 10 psi) was positioned 90 mm from the nozzle and used to populate Bernoulli’s equation to calculate flow velocities:

Eq (1) \begin{equation*}H=\frac{v^2}{2g}\end{equation*}

where *H* is head differential obtained with the pressure gauge (psi), *v* is flow velocity (m s^−1^) and *g* is the gravitational constant (m^2^ s^−1^). With the establishment of the flow velocities, shear strain rates were calculated using the equation:

Eq (2) \begin{equation*}e=\frac{\partial v}{\partial y}\end{equation*}

where *v* is mean jet velocity (m s^−1^) calculated for the different flow rates and *y* is the fine scale resolution of the shear strain at the width of the test specimens, used as the distance perpendicular to the force (Neitzel *et al.,* 2004). Here, *y* was estimated at 1.5 mm (egg diameter; Alix *et al.,* 2015), 1 mm (larvae 12–18 DPH), 2 mm (larvae 28–30 DPH) and 10 mm (juveniles) (Supplementary Table 2).

For exposure to the shear environment, eggs (one to two ribbon fragments), larvae (*n* = 15 for 12–18 DPH and *n* = 10 for 28–30 DPH) or individual fish (juveniles) were inserted directly into the jet via a delivery tube, following Navarro *et al.* (2019). Adults were not tested as they were too large for the delivery tube. Eggs were obtained by cutting a ribbon with scissors into fragments ~30 mm^2^. Each fragment was placed on a petri dish and approximately 25 eggs (Supplementary Table 2) were counted using a microscope. Larvae 12–18 DPH and 28–30 DPH (Supplementary Table 2) were collected from holding aquaria using pipettes into a 50-ml beaker and poured into the delivery tube. Juvenile redfin (Supplementary Table 2) were individually collected from holding tanks and inserted headfirst into the delivery tube (Supplementary Fig. 2). Specimens were flushed from the delivery tube into the flume with a gentle flow of water. For eggs and both larval stages, 5 replicates were exposed to the four shear strain treatments, whereas 10 replicates were used for juveniles (each individual fish was a replicate), with the order randomized. Along with a control of 0 s^−1^, four standardized flow velocities were converted to shear strain for each life stage: 3367–12 353 s^−1^ (eggs), 5050–18 530 s^−1^ (larvae 12–18 DPH), 2525–9265 s^−1^ (larvae 28–30 DPH) and 505–1853 s^−1^ (juveniles) (Supplementary Table 3).

After exposure to a shear event, each egg and larvae replicate was collected from the retrieval net and placed into its own aerated 700-ml plastic jar. Juvenile redfin were retrieved from the net and transferred into perforated baskets in the holding tanks for each replicate. All treatments were accompanied by a set of replicate controls, where fish were passed directly into the retrieval net, bypassing the shear flume. Survival of all fish was assessed immediately and at 24 h after exposure. Eggs were deemed to have survived if movement was observed inside the egg or they hatched and were free swimming. After the 24-h survival assessment, any fish remaining alive were euthanized (100 mg l^−1^ benzocaine).

### Pressure experiments

A custom-built pressure chamber was used to simulate the extreme (7600 kPa) and fast transient (17 ms) pressure changes expected within the PSH during pumping (Supplementary Fig. 3; chamber described in Doyle *et al.,* 2020). The pressure profile represented all six Francis turbines pumping at maximum flow, with the lower reservoir at full supply level and upper reservoir at minimum operational level, and a total travel time of 7133 s (Fig. 2). This profile started with a slow compression (220–1100 kPa over 1997 s) occurring during the transition from the lower reservoir intake to the turbine draft tube. This was immediately followed by an extremely high (1100–7600 kPa) and rapid compression (19 ms), simulating movement from the draft tubes to the turbines. Finally, a slow decompression to 10 kPa simulated the fish moving through to the outlet in the upper reservoir (within 5134 s; Fig. 2).

Test specimens of each life stage were inserted into two cylindrical capsules, following the procedure described in Doyle *et al.* (2020). Capsules were inserted into the pressure chamber and the pressure profile was initiated. Each running of a profile constituted a single replicate, with five replicates of each experimental group (pressure and control) assessed per life stage. Each replicate consisted of approximately 25 redfin eggs, 30 larvae 12–18 DPH, 10 larvae 28–30 DPH, six juveniles and two adults (Supplementary Table 2). Controls consisted of placing each life stage replicate into the chamber and leaving them for the same amount of time but without a pressure change applied. Handling procedures to transfer the test specimens to the pressure chamber and survival assessments were the same as those for the shear tests.

### Statistical analysis for shear and pressure experiments

For the immediate and delayed (24 h) survival of eggs and larvae, the total number of fish was classified binomially as either dead (0) or alive (1). Juveniles (for shear and pressure experiments) and adults (pressure experiments) were assessed individually, and the same binomial distribution was assigned for each fish.

The probability of survival for each life stage was estimated for shear strain and pressure experiments using General Linear Models. Estimated least square means were used to calculate survival probability of each life stage and experimental groups (shear strain rates and control; pressure and control). A log likelihood ratio (LLR) test was used to compare survival probability between experimental groups, and post hoc Scheffe tests were undertaken for main shear effects. When one or more groups had zero or 100% survival across all replicates, a Firth maximum likelihood penalty (Firth, 1993) was applied to the overall model test and the invariant group(s) removed from the post hoc analyses. All statistical analyses were performed using Statistical Analysis Software (SAS Institute, Cary, NC, USA).

### BS modelling

Deterministic and stochastic models were applied to estimate the survival likelihood of each redfin life stage to BS. For both models, the design of the Francis turbine, including number of blades, wicket gate characteristics and revolutions per minute, were input parameters provided by the power company. A deterministic model for Kaplan turbines (Deng *et al.,* 2007; Turnpenny *et al.,* 2000) was adopted to approximate strike probabilities, with a derivation for Francis turbines (for model calculations, see Supplementary Fig. 4). Given the lack of available data on strike injuries for each life stage, survival estimates considered any fish struck by a blade to be mortally injured. Thus, probability of survival was defined as *S = 1*–*P*. The deterministic model was applied at three turbine discharges (minimum, mid-point and maximum flows, as supplied by the hydropower company) and the corresponding wicket gate opening angles. Fish orientation relative to the flow can vary between 0° and 90° and, since the deterministic model predicts a single estimate for each combination of input values, the arithmetic mean 45° was fixed as the orientation model scenarios. Similarly, fish apparent length was fixed at the measured length for each life stage (Supplementary Table 2) multiplied by cos (45°).

Stochastic modelling used the software @RISK (Palisade Corporation, Ithaca, New York), which allows for a range of distributions for all independent variables to be used, so the variability within a particular range is captured in the calculations and predicted in the models. Variables used were wicket gate angle, fish size and orientation in relation to flow. A Monte Carlo simulation (10 000 realizations) was used for the analysis. Wicket gate angle was assigned a uniform distribution ranging from minimum to maximum flow angle, and discharge was linear interpolated using the provided flow information. A uniform distribution (0° to 90°) was assigned for fish orientation in relation to flow, and fish size (Supplementary Table 2) was given a normal distribution. To test the significance of input variables to the output BS predictions of the stochastic model, sensitivity and scenario analyses were performed using @RISK. From this test, the higher the regression coefficient, the greater the contribution of the input variable to the BS prediction.

## Results

### Shear

**Figure 3:**
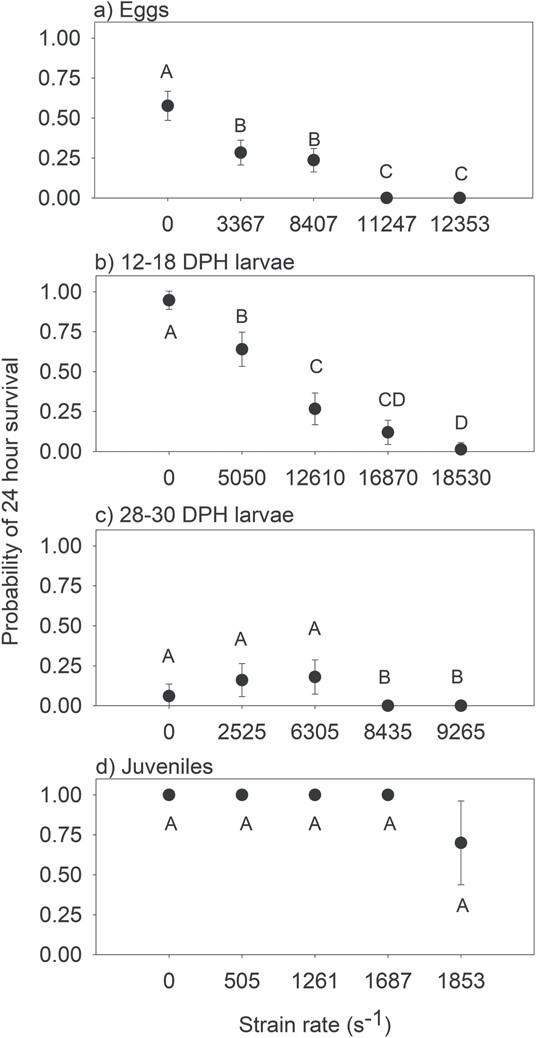
Mean (± 95% CI) probability of 24-h survival of (a) egg, (b) larval 12–18 DPH, (c) larval 28–30 DPH and (d) juvenile life stages of redfin exposed to laboratory-generated shear strain rates. Treatments with different letters are significantly different from one another (*P* < 0.05).

Redfin survival significantly declined as strain rates increased for all life stages tested (Table 1; Fig. 3). Survival of eggs and the earlier larval stage (12–18 DPH) showed the greatest change across the tested strain rates, being relatively high at low strain rates and substantially decreasing as strain rate increased (Fig. 3). The probability of egg survival significantly declined from 0.58 in the controls to 0.24 once strain rate reached 8407 s^−1^, and was zero once strain rates reached 11 247 s^−1^ (Fig. 3). The probability of 12–18 DPH larvae survival was close to 1.0 in the controls, but significantly declined once strain rates reached 5050 s^−1^, then once again at 12610 s^−1^, declining to near zero at the highest strain rate tested (18 530 s^−1^) (Fig. 3). It was more difficult to discern shear-related impacts for the 28–30 DPH larvae, because the control individuals of this life stage had low survival owing to a rapid and unexpected decline in water temperature. Nonetheless, there was still some 28–30 DPH larval survival in all treatments except for the two highest strain treatments 8435 and 9265 s^−1^ (Fig. 3). Juvenile survival probabilities remained at 1.0 until the strain rate reached 1853 s^−1^, where survival probability significantly declined to 0.7 (Fig. 3; Supplementary Table 4).

**Table 1:** Results from the LLR tests used to compare the survival probability between experimental groups for the shear and pressure experiments.

**Life stage**	**Shear**			**Pressure**		
	Df	LLR	p	Df	LLR	p
Eggs	4	181.20	< 0.0001	1	30.44	< 0.0001
12–18 DPH larvae	4	222.04	< 0.0001	1	0.10	0.94
28–30 DPH larvae	4	20.46	< 0.0005	1	1.09	0.29
Juveniles	4	10.48	0.03	100% survival all round		
Adults	Not tested			100% survival all round		

A Firth maximum likelihood penalty (Firth, 1993) was applied to the overall model test for the impact of shear on redfin eggs because one or more groups had 0% survival across all replicates. Adults were not tested for shear impacts as they were too large for the shear flume delivery tube. Also, all juvenile and adult redfin survived the pressure experiments, and therefore were not statistically tested.

### Pressure

**Figure 4:**
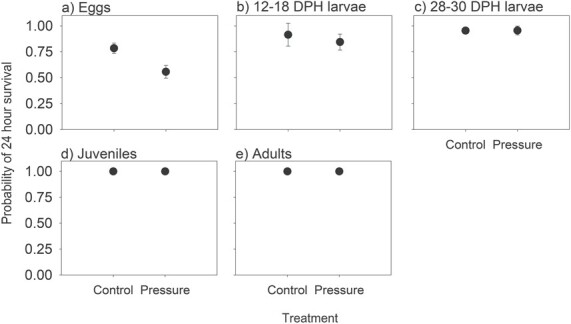
Mean (± 95% CI) probability of 24-h survival of (a) egg, (b) larval 12–18 DPH, (c) larval 28–30 DPH, (d) juvenile and (e) adult life stages of redfin exposed to the pressure profile expected to occur through the PSH (Fig. 2). Significant differences in the estimated mean survival between pressure and control groups were observed for redfin perch eggs (*P* < 0.0001) only.

The probability of survival of both eggs and 12–18 DPH larvae following the simulated PSH pressure profile was lower than their respective controls (Fig. 4; Supplementary Table 5), although the difference was only statistically significant for eggs (Table 1). All juvenile and adult redfin, and more than 0.95 of the 28–30 DPH larvae, survived the simulated pressure profile, indicating that there was no difference with the controls (Fig. 4).

### Blade strike

High probability of survival was predicted across all redfin life stages using both models (Table 2). Deterministic modelling predicted eggs and both larval stages were extremely unlikely to be struck by the Francis turbine blade, irrespective of the flow discharge (Table 2). For these life stages, predicted BS ranged from 0.23% for eggs at minimum turbine flow to 1.70% for the 28–30 DPH larvae at mid-point turbine flow. Consequently, the predicted survival rates were also very high for the same scenario, with 99.77% and 98.3% for eggs and 28–30 DPH larvae, respectively (Table 2). A higher probability of BS was predicted for juveniles and adults. BS predictions for juveniles to be struck ranged from 11.7% to 17.1% and 20% to 28.8% for adults for scenarios where the turbines would be operating at minimum and mid-point flow, respectively. The corresponding probability of survival was predicted at 88.3% to 82.9% for juveniles and 80% to 71.2% for adults (Table 2).

**Table 2:** Predictions of BS using two predictor models: deterministic and stochastic

***Deterministic model***										***Stochastic model***		
Turbine Flow range	Min flow			Mid flow			Max flow			Predictor variables regression coefficient		
Fish Length range^a^	Min	Mean	Max	Min	Mean	Max	Min	Mean	Max		Fish length^a^	Wicket gate angle
***Eggs***												
BS	0.23	0.24	0.25	0.25	0.26	0.27	0.23	0.24	0.25	0.25	0.66	0.52
S	99.77	99.76	99.75	99.75	99.74	99.73	99.77	99.76	99.75	99.75		
***Larvae 12–18 DPH***												
BS	0.50	0.70	0.90	0.50	0.80	1.00	0.50	0.70	0.90	0.57	1.00	0.03
S	99.50	99.30	99.10	99.50	99.20	99.00	99.50	99.30	99.10	99.43		
***Larvae 28–30 DPH***												
BS	0.90	1.20	1.60	0.90	1.30	1.70	0.90	1.20	1.60	0.92	1.00	0.03
S	99.10	98.80	98.40	99.10	98.70	98.30	99.10	98.80	98.40	99.08		
***Juvenile***												
BS	11.70	13.90	15.90	12.50	14.90	17.10	11.70	14.00	16.00	10.30	1.00	0.03
S	88.30	86.10	84.10	87.50	85.10	82.90	88.30	86.00	84.00	89.70		
***Adult***												
BS	20.00	23.10	26.90	21.40	24.70	28.80	20.10	23.20	27.00	17.31	1.00	0.03
S	80.00	76.90	73.10	78.60	75.30	71.20	79.90	76.80	73.00	82.69		

For both models, the mean probability of BS and associated mean probability of survival (S) are presented for each life stage of redfin for a Francis turbine designed for the PSH. The deterministic model includes the range of discharges (minimum, mid-point and maximum flow) and fish length. The stochastic model has two predictor variables, fish length and wicket gate angle, presented here with the regression co-efficient.

a For fish length range used in the BS model, refer to Supplementary Table 2.

Similar results were obtained using stochastic modelling. The predicted probability of BS ranged from 0.25% for eggs to 17.31% for adults (Table 2). As such, probability of survival was also high, with 82.69% of adults (the most susceptible life stage to BS) predicted to survive when passing the turbine blades (Table 2). Sensitivity analyses conducted for the stochastic model showed that fish length was the main variable contributing to the BS probability for all life stages (*r* = 1.00), except for eggs (Table 2). For the latter, fish length (*r* = 0.66) and wicket gate angle (*r* = 0.52) contributed similarly to the BS predictions.

## Discussion

Our results indicate that redfin survival could be high when exposed to the hydraulic (shear and extreme compression) and physical (BS) stressors expected to be experienced in a 2000-MW PSH during its pumping phase, but there would be ontogenetic differences in survival rates. While extreme compression had minimal impact on any life stages, earlier life stages had markedly reduced survival rates under the higher shear strain rates tested, whereas BS was more likely to affect the survival of older life stages because of their larger size.

### Shear

A large proportion of redfin (eggs, larvae and juveniles) survived exposure to the elevated shear strain expected during PSH passage. Our results, alongside those of other studies (Deng *et al.,* 2010; Neitzel *et al.,* 2004) support the notion that fish survival will typically decrease as shear strain increases. Juvenile redfin appear relatively tolerant to shear, with their survival rates declining once levels reached 1853 s^−1^. In comparison, juvenile *Oncorhynchus* salmonids survival reduced at shear strains ranging from 734–1008 s^−1^, while American shad (*Alosa sapidissima*) were affected once shear strains exceeded 400 s^−1^ (Neitzel *et al.,* 2004). Iridescent sharks (*Pangasianodon hypophthalmus*) appear slightly more tolerant to shear, only being impacted once shear strains reached 1185 s^−1^ (Colotelo *et al.,* 2018).

Susceptibility to higher strain rates was more evident in the earlier life stages of redfin (eggs and 12–18 DPH larvae) than in the later life stages (28–30 DPH larvae and juveniles). Navarro *et al*. (2019), although testing a lower range of strain rates, also observed a relatively lesser shear impact on the older life stages of Australian perciformes than on their younger life stages. This could be attributed to several factors, including the development of a more robust integument and internal organs in the later life stages (Navarro *et al.,* 2019) and the magnitude of shear forces that dominate the environment of the smaller-sized eggs and larval stages.

### Pressure

Most redfin eggs, larvae, juveniles and adults would likely survive the extreme compression (to ~7600 kPa) expected during the pumping phase of the PSH. Such extreme and rapid pressurization has rarely been assessed since conventional hydropower lends itself to rapid decompression instead (Brown *et al.,* 2014). Unlike conventional hydropower studies where decompression causes significant barotrauma-related injuries and mortality (Brown *et al.,* 2014), extreme compression had little impact on overall survival for all life stages. Of the studies published, high survival has been reported for juvenile and adult redfin, whitefish (*Coregoma* sp.), rainbow trout, grayling (*Thymallus* sp*.*), carp (*Cyprinus carpio*) and roach (*Leuciscus rutilus*) when exposed to a rapid increase in pressure (5000 kPa) in a hydrostatic pressure study (Lampert, 1976). Under the same experimental conditions as the study presented here, mosquito fish (*Gambusia holbrooki)* also had high survival to extreme compression (Doyle *et al.,* 2020). Rainbow trout have also been exposed to rapid (i.e. <1 min) pressure rises (to 1277 kPa) followed by a sudden pressure release (Rowley, 1955). The trout were immobilized while under pressure but resumed normal activity following pressure release, which was similar to our observations for redfin and those in Doyle *et al.* (2020) for mosquitofish. These responses are likely because the swim bladder, the most susceptible organ to barotrauma, compresses and decreases in size. Studies reporting pressure-related impacts on fish relating to conventional hydropower do so when the swim bladder expands during decompression. Thus, the pumping mode of PSH is initiating a different physiological response than conventional hydropower.

### Blade strike

Our BS model results indicate that most redfin will avoid a strike from a turbine blade during passage through the PSH facility. These results concur with the generally accepted concept that BS is related to fish size, with larger fish being more likely to be struck by a rotating blade (Ferguson *et al.,* 2008; Romero-Gomez and Richmond, 2014; Schweizer *et al.,* 2012). For fish eggs and small fish, which have relatively large surface area-to-mass ratios, their probabilities of being stuck could be even lower than reported here as they could be pulled around, rather than colliding with, the blade edge (Deng *et al.,* 2007).

Our models assumed that all strikes would lead to mortality, but in reality this is not the case, and as such a ‘mutilation ratio’ is used to correct for this effect (Von Raben, 1957). Because there was no ‘mutilation ratio’ available for redfin, our results likely underestimated survival rates. Since survival predictions were high regardless, we do not expect any underestimation would change how the results are interpreted. Final designs for the PSH we simulated are still being refined. As more accurate and detailed turbine geometry and flow rates for the PSH become available, it would be possible to refine the models further. However, based on the high survival rates predicted, vastly conflicting results would not be expected.

### Further considerations

Our study provides a representation of what might happen when a fish passes a PSH facility during its pumping phase. Nonetheless, it is worth considering how the test conditions may differ from a real-world passage scenario. Firstly, we examined the effects of shear, pressure and BS independently of one another. There is a possibility that in a real-world passage scenario, these stressors will affect a fish cumulatively (Čada, 2001). For example, a fish disoriented following exposure to shear stress (Groves, 1972) could become more vulnerable to BS, resulting in a lower probability of survival. Similarly, a fish under extreme pressure has little control over its movement and therefore may be more susceptible to strike. We observed fish became negatively buoyant once compressed, so these fish would generally be unable to maintain their position in the water column and have reduced swimming capacity (Odeh *et al.,* 2002) while passing through the turbine.

Secondly, exposure to injurious levels of shear and pressure or a strike from a blade could result in injuries (e.g. abrasions, internal bleeding and spinal damage) that may not lead to immediate mortality. As such, these injuries may ultimately reduce fitness and could result in delayed mortality (Čada, 2001; Navarro *et al.,* 2019) or a greater susceptibility to ecological processes such as predation following passage (Schilt, 2007). This mortality would not have been accounted for in our shear and pressure experiments, as we only held fish for 24 h post-experimentation.

Finally, we simulated the most extreme operating scenario being proposed for the PSH, that is, all six turbines operating. Most of the time, the PSH will not be operated in such an extreme fashion, particularly when less turbines are operating. Although the maximum and minimum pressure values remain similar whether all or some turbines are operating during pumping, the rates of change between maximum and minimum pressure values will be far slower when fewer turbines are in operation. Furthermore, not all fish passing through hydropower turbines are exposed to damaging levels of shear or BS as this depends on the route of passage through the system (Deng *et al.,* 2011). Actual impacts on fish populations will depend on the total number or proportion of fish passing through areas with lethal or injurious stressors, the final operating conditions of the PSH facility and the cumulative effects of all stressors (Neitzel *et al.,* 2004).

Independent assessments of hydraulic and physical stressors provide a useful indicator of redfin survival through the PSH. However, the long-term success of the species that is transferred through the PSH facility will not only depend on passage survival. The likelihood of fish becoming entrained at the lower reservoir (Rytwinski *et al.,* 2017) and the survival and persistence of the species in the upper reservoir (Coutant and Whitney, 2000; Grimaldo *et al.,* 2009) are also other important considerations. While passage studies like ours are an important part of the story, they should be viewed alongside studies into entrainment risk and habitat suitability. Ultimately, accurately assessing the survival of turbine-passed fish will require field evaluations (Čada, 1990). Field evaluations could empirically examine and quantify upstream/downstream fish propagule pressure and post-passage movement propensity from a PSH facility.

### Implications for conservation

Although our study tested a single species and simulated one specific PSH facility, our results draw attention to the need to consider two opposing management implications for the successful transfer of aquatic species through PSH facilities: (i) species population conservation that aims to maximize the survival of a desirable species; and (ii) the risk of the unintentional transfer of an undesirable species, as is true for redfin in Australia. In the latter scenario, fish entrainment could potentially be prevented to minimize the transfer of undesirable species or reduce fish losses from connected waterways by exploring mitigation options such as screens (Andrade *et al.,* 2012) or non-physical barriers like light, sound or electrical devices (Nestler *et al.,* 1992; Schilt, 2007). However, these measures have yet to be demonstrated to be 100% effective, practical and/or acceptable to regulatory authorities for such large developments (Čada and Sale, 1993; Ferguson, 1992). Since we have shown that all life stages will likely survive passage, our data has implications for the type of barrier technology that might be designed to consider all life stages. Exclusion devices may minimize entrainment of undesirable species, but they may create issues in severing population connectivity of desirable species; hence management options should be prioritized and tailored to the individual PSH facility.

## Conclusion

The global surge of water infrastructure developments has meant that fish and aquatic biota are now increasingly facing physical and hydraulic stressors at magnitudes well beyond their physiological adaptations. New PSH and conventional hydropower facilities will undoubtedly contribute to this infrastructure surge, making it important to consider the trade-off between achieving more ambitious renewable energy targets and conserving the integrity of freshwater ecosystems. Proposing general conservation actions for the varying types of hydropower operations hoping that these will be a ‘one-size-fits-all’ approach is insufficient. Where possible, the entire life history of the fish population of interest should be studied to inform construction, operation and policy decisions for the development of either fish protection or passage measures, or to reduce or mitigate the risk of unwanted aquatic species transfers. We encourage the replication and consideration of the concepts presented in this study to be applied to other aquatic species, whether they are of conservation value or undesirable, to ensure that these species are accurately represented in hydropower policies and environmental assessments.

## Funding

This research was supported by Snowy Hydro Limited with additional support from New South Wales Department of Primary Industries and Charles Sturt University. The work was funded by Snowy Hydro Limited but gives an independent and scientific account of potential upgrade works.

## Data availability

The data underlying this article cannot be shared publicly because the hydropower developer that funded this study has strict confidentiality requirements. The data may be shared on reasonable request to the corresponding author if the hydropower developer approves.

## Supplementary Material

Web_Material_coac017
