# Supplementary material for: Survival estimates across five life stages of redfin (Perca fluviatilis) exposed to simulated pumped-storage hydropower stressors
Source: Conserv Physiol. 2022 Apr 20;10(1):coac017. doi: 10.1093/conphys/coac017 (PMC9041421; doi:10.1093/conphys/coac017)
Supplement: Web_Material_coac017 [file web_material_coac017.docx]

**Tables**

**Table 1:** Results from the log likelihood ratio (LLR) tests used to compare the survival probability between experimental groups for the shear and pressure experiments. A Firth maximum likelihood penalty (Firth 1993) was applied to the overall model test for the impact of shear on redfin eggs because one or more groups had zero percent survival across all replicates. Adults were not tested for shear impacts as they were too large for the shear flume delivery tube. Also, all juvenile and adult redfin survived the pressure experiments, and therefore were not statistically tested.

| **Life stage** | **Shear** | |  | **Pressure** | |  |
| --- | --- | --- | --- | --- | --- | --- |
|  | Df | LLR | p | Df | LLR | p |
| Eggs | 4 | 181.20 | < 0.0001 | 1 | 30.44 | < 0.0001 |
| 12-18 DPH larvae | 4 | 222.04 | < 0.0001 | 1 | 0.10 | 0.94 |
| 28-30 DPH larvae | 4 | 20.46 | < 0.0005 | 1 | 1.09 | 0.29 |
| Juveniles | 4 | 10.48 | 0.03 | 100% survival all round | | |
| Adults | Not tested | |  | 100% survival all round | | |

**Table 2:** Predictions of blade strike using two predictor models; deterministic and stochastic. For both models, the mean probability of blade strike (BS) and associated mean probability of survival (S) are presented for each life stage of redfin for a Francis turbine designed for the PSH. The deterministic model includes the range of discharges (minimum, mid-point and maximum flow) and fish length. The stochastic model has two predictor variables, fish length and wicket gate angle, presented here with the regression co-efficient.

| ***Deterministic model*** | | | | | | | | | | ***Stochastic model*** | | |
| --- | --- | --- | --- | --- | --- | --- | --- | --- | --- | --- | --- | --- |
| Turbine Flow range | Min flow | | | Mid flow | | | Max flow | | |  | Predictor variables regression coefficient | |
| Fish Length range^ | Min | Mean | Max | Min | Mean | Max | Min | Mean | Max |  | Fish length^ | Wicket gate angle |
| ***Eggs*** | | | | | | | | | |  |  |  |
| BS | 0.23 | 0.24 | 0.25 | 0.25 | 0.26 | 0.27 | 0.23 | 0.24 | 0.25 | 0.25 | 0.66 | 0.52 |
| S | 99.77 | 99.76 | 99.75 | 99.75 | 99.74 | 99.73 | 99.77 | 99.76 | 99.75 | 99.75 |  |  |
| ***Larvae 12 – 18 DPH*** | | | | | | | | | |  |  |  |
| BS | 0.50 | 0.70 | 0.90 | 0.50 | 0.80 | 1.00 | 0.50 | 0.70 | 0.90 | 0.57 | 1.00 | 0.03 |
| S | 99.50 | 99.30 | 99.10 | 99.50 | 99.20 | 99.00 | 99.50 | 99.30 | 99.10 | 99.43 |  |  |
| ***Larvae 28 – 30 DPH*** | | | | | | | | | |  |  |  |
| BS | 0.90 | 1.20 | 1.60 | 0.90 | 1.30 | 1.70 | 0.90 | 1.20 | 1.60 | 0.92 | 1.00 | 0.03 |
| S | 99.10 | 98.80 | 98.40 | 99.10 | 98.70 | 98.30 | 99.10 | 98.80 | 98.40 | 99.08 |  |  |
| ***Juvenile*** | | | | | | | | | |  |  |  |
| BS | 11.70 | 13.90 | 15.90 | 12.50 | 14.90 | 17.10 | 11.70 | 14.00 | 16.00 | 10.30 | 1.00 | 0.03 |
| S | 88.30 | 86.10 | 84.10 | 87.50 | 85.10 | 82.90 | 88.30 | 86.00 | 84.00 | 89.70 |  |  |
| ***Adult*** | | | | | | | | | |  |  |  |
| BS | 20.00 | 23.10 | 26.90 | 21.40 | 24.70 | 28.80 | 20.10 | 23.20 | 27.00 | 17.31 | 1.00 | 0.03 |
| S | 80.00 | 76.90 | 73.10 | 78.60 | 75.30 | 71.20 | 79.90 | 76.80 | 73.00 | 82.69 |  |  |

^For fish length range used in the blade strike model refer to Supplementary Table 2.

**Figures**


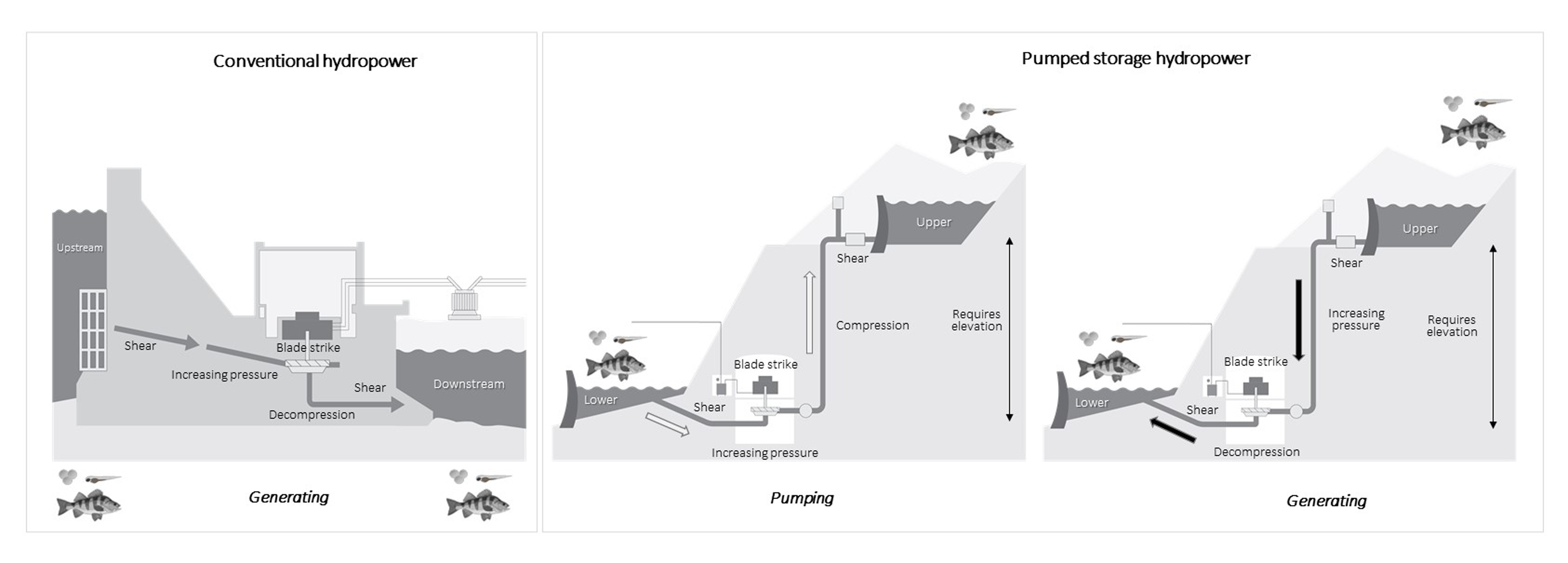


**Figure 1:** Schematic of a conventional hydropower facility (left) and a PSH facility during pumping (middle) and power generation phases (right), and showing the different hydraulic (decompression, compression, shear) and physical (blade strike) stressors entrained fish, eggs and larvae may be exposed to. Arrows indicate water flow and fish direction through each facility. For the conventional scenario, Upstream and Downstream depict the river flow direction. For the PSH scenario, Lower refers to the lower reservoir and Upper is the upper reservoir. Diagram not to scale.


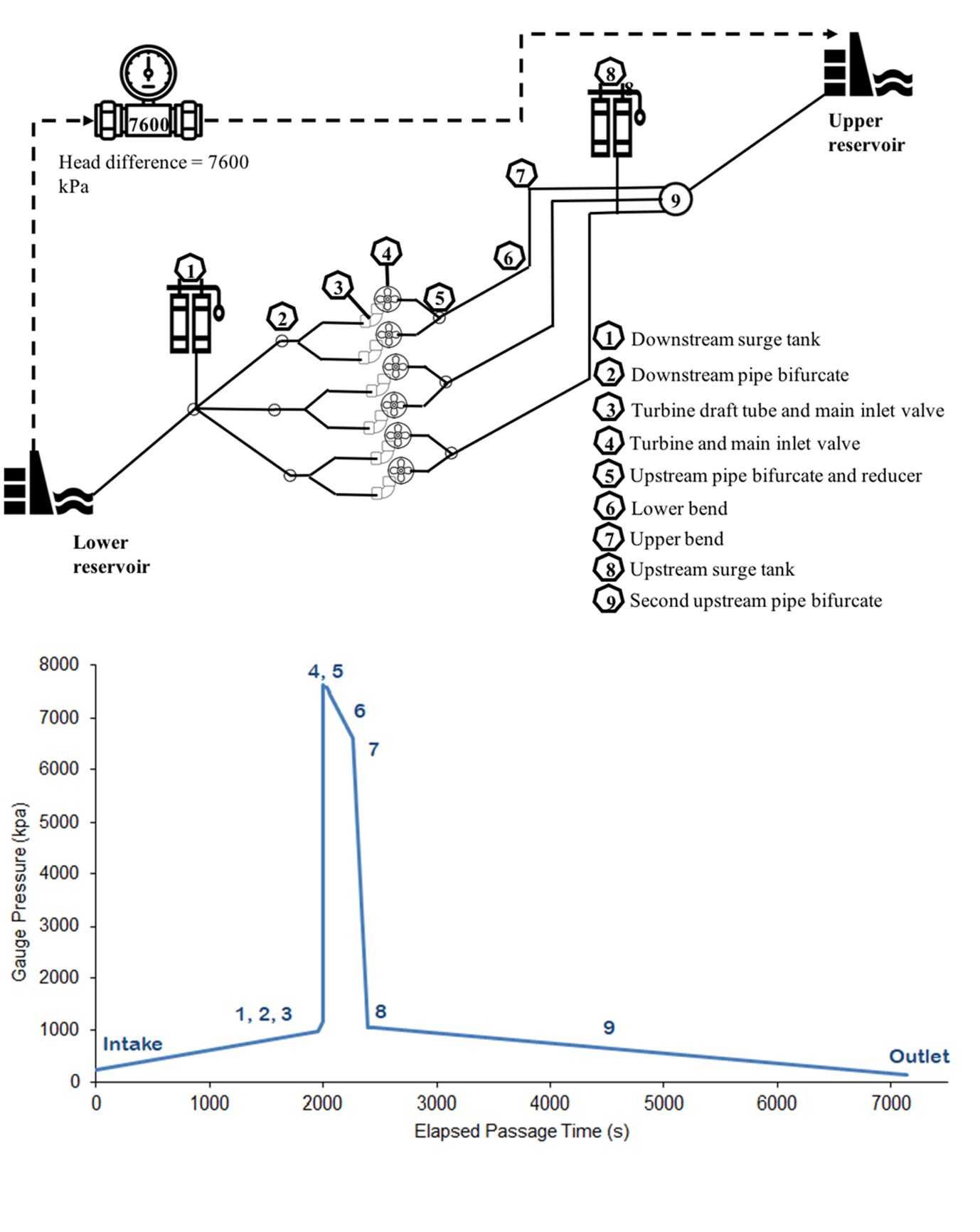


**Figure 2:** Schematic of the PSH (top) and the expected pressure profile (bottom) generated during full pumping capacity (i.e. six turbines operating). Numbers 1–9 identify different locations in the facility. The head difference reflects the lower reservoir operating at full supply level and upper reservoir at minimum operating level.


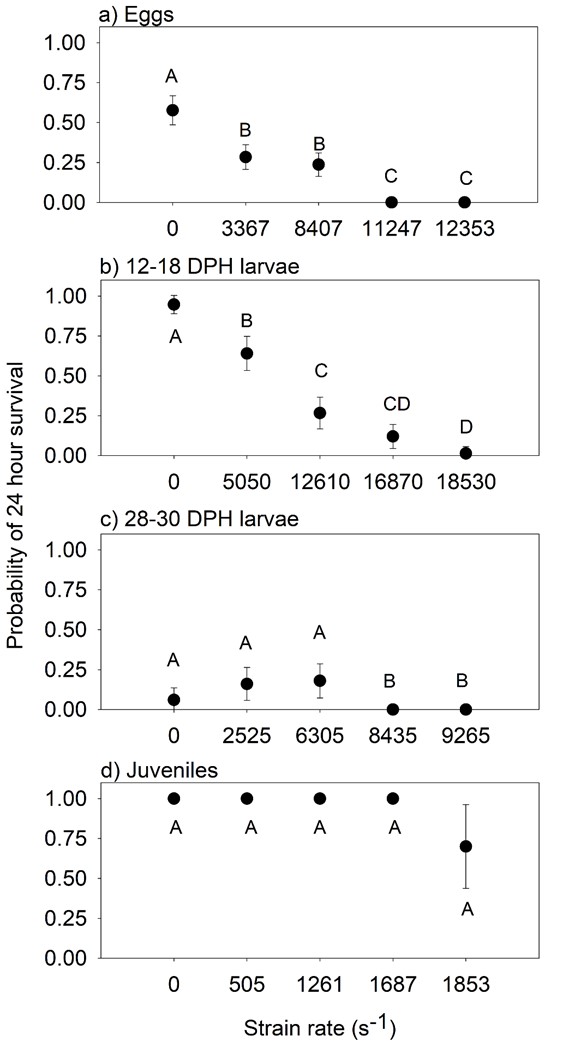


**Figure 3:** Mean (± 95% CI) probability of 24-hour survival of (a) egg, (b) larval 12­-18 DPH, (c) larval 28-30 DPH and (d) juvenile life stages of redfin exposed to laboratory-generated shear strain rates. DPH – days post-hatch. Treatments with different letters are significantly different from one another (p < 0.05).

**
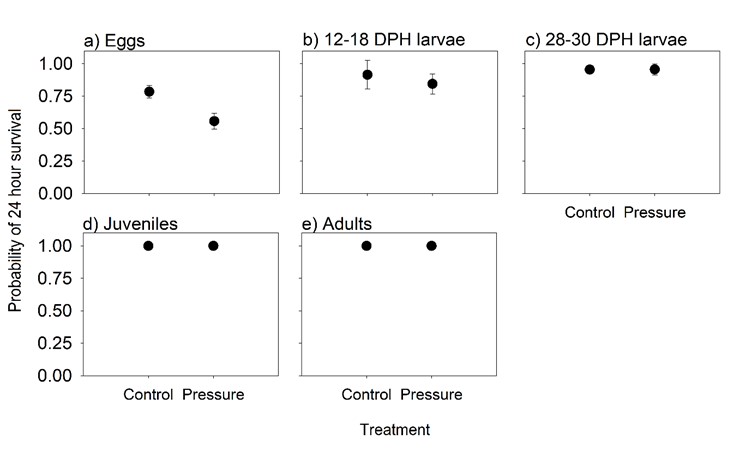
Figure 4:** Mean (± 95% CI) probability of 24-hour survival of (a) egg, (b) larval 12­-18 DPH, (c) larval 28-30 DPH, (d) juvenile and e) adult life stages of redfin exposed to the pressure profile expected to occur through the PSH (Fig. 2). Significant differences in the estimated mean survival between pressure and control groups were observed for redfin perch eggs (p < 0.0001) only. DPH – days post-hatch.

**Supplementary material**

**Supplementary tables**

**Supplementary Table 1:** Water quality parameters for juvenile, adult and brood stock redfin holding tanks. Water quality was measured using a Horiba U-54 (www.horiba.com/en_en/) multi-parameter probe (temperature, pH, dissolved oxygen (%, mg.L^-1^) conductivity (mS.cm^-1^)) and an API^®^ Master Freshwater Test Kit (apifishcare.com) (nitrite, nitrate, total ammonia).

| **Parameter** | **Mean (± SE) and range** | **Mean (± SE) and range** |
| --- | --- | --- |
|  | ***Juvenile and adult redfin*** | ***Brood stock redfin*** |
| Temperature °C | 11.12 ± 0.016 (6.73 – 16.22) | 12.28 ± 0.060 (9.28 – 16.03) |
| pH | 7.6 ± 0.001 (7.02 – 7.86) | 7.61 ± 0.004 (7.21 – 7.76) |
| Conductivity (mS cm^-1^) | 6.4 ± 0.016 (0.07 – 11.4) | 4.80 ± 0.035 (2.54 – 5.51) |
| Dissolved oxygen (mg L^-1^) | 10.15 ± 0.008 (5.91 – 13.05) | 9.63 ± 0.025 (6.67 – 10.57) |
| Total Dissolved Gas Saturation (%) | 97.09 ± 0.074 (56.8 – 119) | 94.39 ± 0.266 (65 – 104.8) |
| Turbidity (NTU) | Always 0 | 0.14 ± 0.026 (0 – 3.7) |
| Nitrite | 0.05 ± 0.001 (0 – 2) | 0.03 ± 0.004 (0 – 0.5) |
| Nitrate | 0.18 ± 0.007 (0 – 5) | Always 0 |
| Ammonia | 2.01 ± 0.010 (0 – 4) | 2.19 ± 0.050 (0.25 – 4) |

**Supplementary Table 2:** Summary of redfin biometrics and number used for each replicate for each experiment (shear, pressure and blade strike) and life stages (eggs, larvae, juveniles and adults). Fish sizes are presented in mm.

| **Experiment** | **Life stage** | **Fish size (mean ± SD, range)** | **No. per replicate** |
| --- | --- | --- | --- |
| *Shear* | Eggs | 24.4 ± 4.4, (19 – 34) * | 1 – 2 fragments |
|  | Larvae 12 - 18 DPH | 5.8 ± 0.6 (4.1 – 7.3) ^ | 15 |
|  | Larvae 28 - 30 DPH | 10.1 ± 1.5 (6 – 14) ^ | 10 |
|  | Juvenile | 115.6 ± 6.5 (98 – 127) ^◊^ | 1 |
| *Pressure* | Eggs | 25.9 ± 4.7 (20 – 35) * | 1 – 2 fragments |
|  | Larvae 12 - 18 DPH | 7.0 ± 1.1 (4.5 – 9.5) ^ | 30 |
|  | Larvae 28 - 30 DPH | 9.7 ± 1.1 (7 – 13) ^ | 10 |
|  | Juvenile | 114.1 ± 6.5 (96 – 131) ^◊^ | 6 |
|  | Adult | 189.5 ± 19.1 (164 – 221)^◊^ | 2 |
|  | Eggs | 1.4 ± 0.03 (1.34 – 1.46) ** |  |
|  | Larvae 12 - 18 DPH | 7.0 ± 1.1 (4.5 – 9.5) ^ |  |
| *Blade strike* | Larvae 28 - 30 DPH | 9.7 ± 1.1 (7 – 13) ^ |  |
|  | Juvenile | 114.1 ± 6.5 (96 – 131) ^◊^ |  |
|  | Adult | 189.5 ± 19.1 (164 – 221)^◊^ |  |

* Number of eggs, counted as the total number inserted in the experimental apparatus on each replicate

**For blade strike modelling the egg diameter was used as the size component

^Larvae were measured by their total length

^◊^Juveniles and adults were measured by their fork length

**Supplementary Table 3:** Shear flume flow rates, mean jet velocities and shear strain rates calculated based on estimated *y* (distance perpendicular to the flow width) for each redfin life stage tested. A control treatment was included for each life stage, but not exposed to flow.

| Life stage | *y* (mm) | Chamber flow rate (L s^-1^) | Mean jet velocity at the nozzle (m s^-1^) | Shear strain rate (1 s^-1^) |
| --- | --- | --- | --- | --- |
|  |  |  |  |  |
| Eggs | 1.5 | 0  12 | 0  5.05 | 0  3367 |
|  |  | 25 | 12.61 | 8407 |
|  |  | 34 | 16.87 | 11247 |
|  |  | 40 | 18.53 | 12353 |
|  |  |  |  |  |
|  |  | 0 | 0 | 0 |
| Larvae 12-18 DPH | 1 | 12 | 5.05 | 5050 |
|  |  | 25 | 12.61 | 12610 |
|  |  | 34 | 16.87 | 16870 |
|  |  | 40 | 18.53 | 18530 |
|  |  |  |  |  |
|  |  | 0 | 0 | 0 |
| Larvae 28-30 DPH | 2 | 12 | 5.05 | 2525 |
|  |  | 25 | 12.61 | 6305 |
|  |  | 34 | 16.87 | 8435 |
|  |  | 40 | 18.53 | 9265 |
|  |  |  |  |  |
|  |  | 0 | 0 | 0 |
| Juveniles | 10 | 12 | 5.05 | 505 |
|  |  | 25 | 12.61 | 1261 |
|  |  | 34 | 16.87 | 1687 |
|  |  | 40 | 18.53 | 1853 |

**Supplementary Table 4:** Results from General Linear Models (GLM) and pairwise comparisons of the estimated survival probability of redfin for each life stage and for each shear strain treatment. Significant differences (p < 0.05*)* in survival between shear strain groups are in bold font.

|  | **Observation** | **Shear strain** | **Shear strain** | **Adjusted p value** |
| --- | --- | --- | --- | --- |
| ***Eggs*** | 1 | 3367 | 0 | **<0.0001** |
|  | 2 | 8407 | 0 | **<0.0001** |
|  | 3 | 8407 | 3367 | 0.6923 |
|  |  |  |  |  |
| ***12-18 DPH*** | 1 | 5050 | 0 | **0.0025** |
|  | 2 | 12610 | 0 | **<0.0001** |
|  | 3 | 16870 | 0 | **<0.0001** |
|  | 4 | 18530 | 0 | **<0.0001** |
|  | 5 | 12610 | 5050 | **0.0005** |
|  | 6 | 16870 | 5050 | **<0.0001** |
|  | 7 | 18530 | 5050 | **0.0002** |
|  | 8 | 16870 | 12610 | 0.2927 |
|  | 9 | 18530 | 12610 | **0.0401** |
|  | 10 | 18530 | 16870 | 0.3208 |
|  |  |  |  |  |
| ***28-30 DPH*** | 1 | 2525 | 0 | 0.3051 |
|  | 2 | 6305 | 0 | 0.2109 |
|  | 3 | 6305 | 2525 | 0.9652 |
|  |  |  |  |  |
| ***Juvenile*** | 1 | 505 | 0 | 1.0000 |
|  | 2 | 1261 | 0 | 1.0000 |
|  | 3 | 1687 | 0 | 1.0000 |
|  | 4 | 1853 | 0 | 1.0000 |
|  | 5 | 1261 | 505 | 1.0000 |
|  | 6 | 1687 | 505 | 1.0000 |
|  | 7 | 1853 | 505 | 1.0000 |
|  | 8 | 1687 | 1261 | 1.0000 |
|  | 9 | 1853 | 1261 | 1.0000 |
|  | 10 | 1853 | 1687 | 1.0000 |

**Supplementary Table 5:** Estimated survival probability (column ‘Survival’) from General Linear Models (GLM) for each life stage of redfin for pressure and control treatments.

| **Life Stage** | **Treatment** | **Pred** | **Uclm** | **Lclm** | **Survival** |
| --- | --- | --- | --- | --- | --- |
| ***Eggs*** | Control | 0.78409 | 0.82961 | 0.73037 | 0.78409 |
|  | Pressure | 0.55731 | 0.61736 | 0.49555 | 0.55731 |
| ***12DPH*** | Control | 0.91489 | 0.97401 | 0.75514 | 0.91489 |
|  | Pressure | 0.84444 | 0.90652 | 0.75241 | 0.84444 |
| ***28DPH*** | Control | 0.95413 | 0.97295 | 0.92325 | 0.95413 |
|  | Pressure | 0.95588 | 0.98201 | 0.89581 | 0.95588 |
| ***Juvenile*** | Control | 1.00000 |  |  | 1.00000 |
|  | Pressure | 1.00000 |  |  | 1.00000 |
| ***Adult*** | Control | 1.00000 |  |  | 1.00000 |
|  | Pressure | 1.00000 |  |  | 1.00000 |

**Supplementary figures**


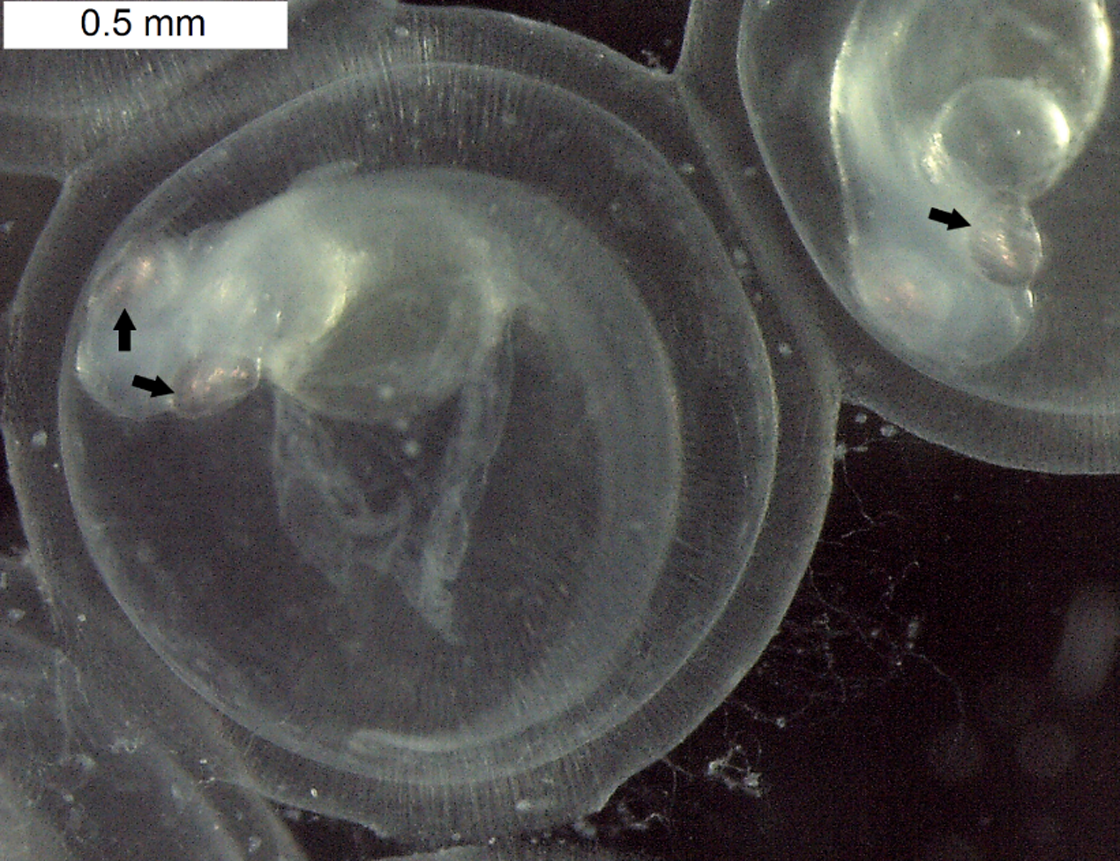


**Supplementary Figure 1:** Redfin embryo at the development stage where eyes are visible (black arrows) and wriggling movements were initiated. This is the life stage used for the ‘egg’ experiments for shear and pressure experiments and measurements were supplied for blade strike models.


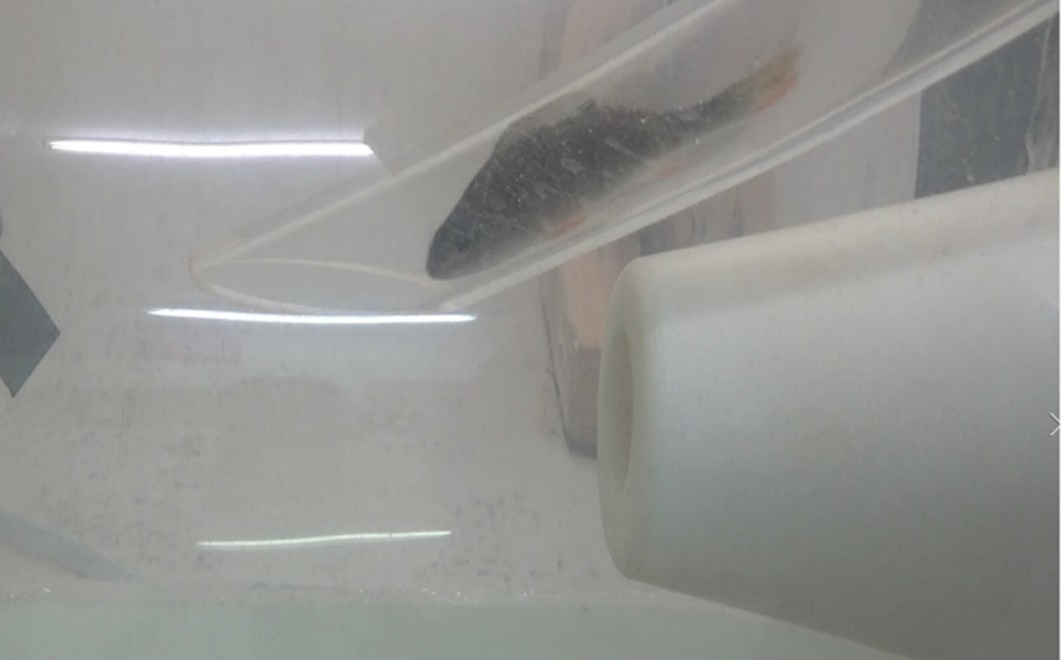


**Supplementary Figure 2:** A juvenile redfin entering the conical nozzle of the shear flume used to reduce the diameter of the flow, effectively accelerating the flow to generate a shear environment.


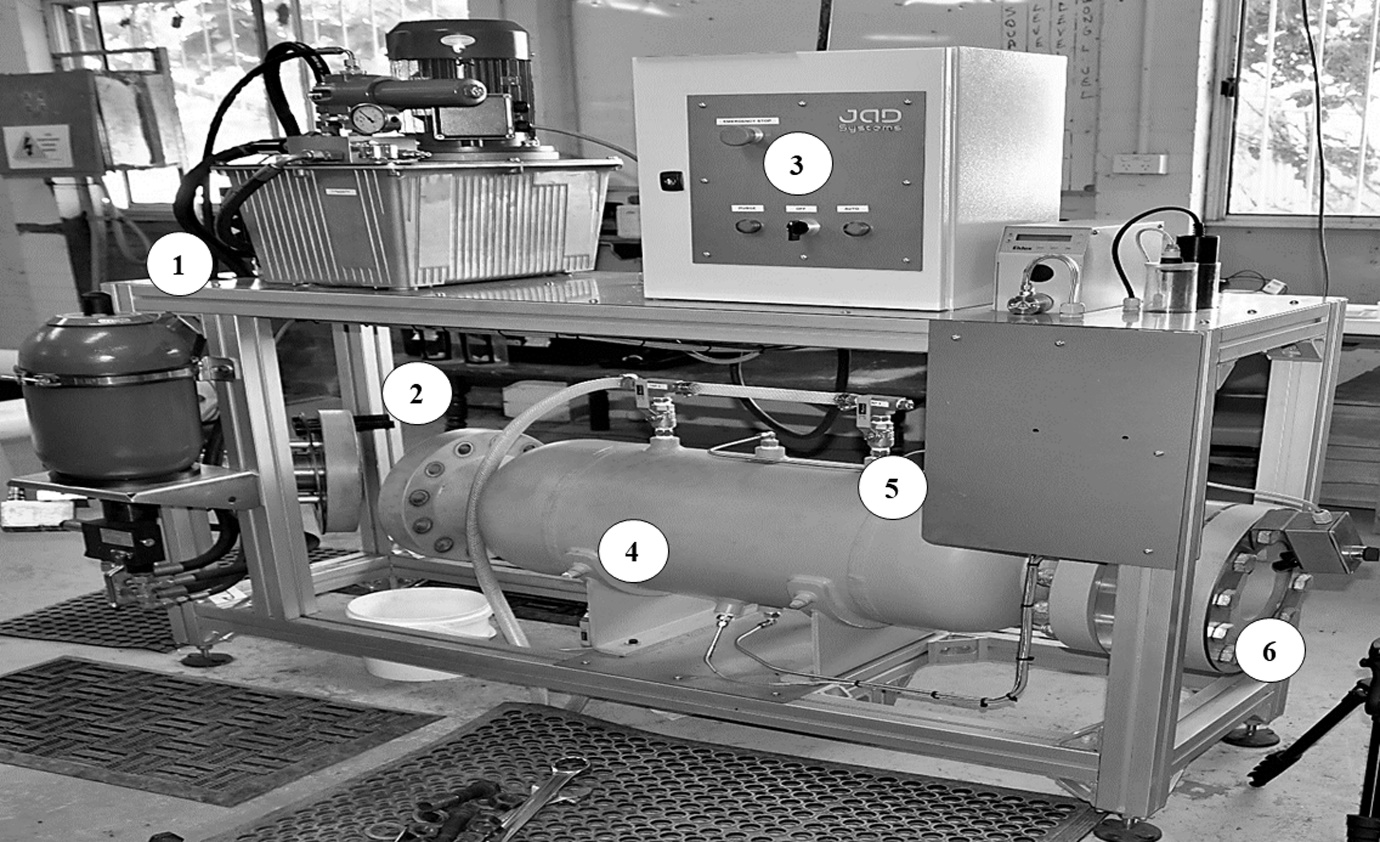


**Supplementary Figure 3:** Diagram of the pressure chamber and labelled components 1) hydraulic pump and cylinder, 2) removable flange where fish capsules were inserted, 3) control panel, 4) pressure vessel, 5) pressure sensor and 6) fixed viewing window.


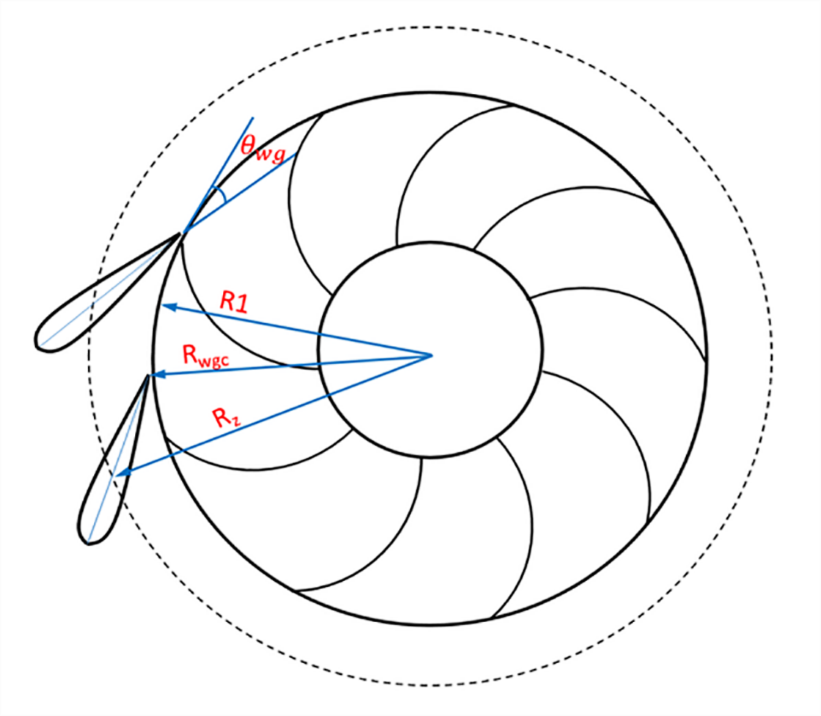


**Supplementary Figure 4:** Schematic showing the wicket gate angle and radius of the imaginary cylinder of the wicket gate exit.

**Supplementary blade strike model calculations**

Initially, the surface of an imaginary cylinder of a Francis turbine wicket gate exit (Supplementary Fig. 4) was calculated as:

$A_{wgc}=2\pi R_{wgc}h_{wg}$ Eq (1)

where $R_{wgc}$ is the imaginary cylinder radius and $h_{wg}$is wicket gate height. Given the high head (> 600 m) for the turbine and low specific speed, the turbine runner leading edge cylinder is expected to be adjacent to the imaginary cylinder of the wicket gate exit and, therefore, $R_{wgc}=R_{1}$, where $R_{1}$is the turbine runner leading edge radius. In this case, the radial velocity at the wicket gate exit is described by:

$V_{r}=\frac{Q}{A_{wgc}}=\frac{Q}{2\pi R_{wgc}h_{wg}}= \frac{Q}{2\pi R_{1}h_{wg}}$ Eq (2)

Knowing the radial velocity, Deng *et al.,* (2007) defined the time between sweeps of two successive blades as ‘critical passage time’ ($t_{cr}$), which is the time for a fish to pass through the plane of the leading edges of the blades. Thus, if a fish approaches the wicket gate exit at the same angle (θ) as the wicket gate opening, the time for a fish to pass through the imaginary cylinder of the turbine runner leading edges is:

$t=\frac{l\cdot sin\theta}{V_{r}}$ Eq (3)

where $l$ is fish length, so $t_{cr}$ is expressed as:

$t_{cr}= \frac{1}{n\cdot(\frac{N}{60})}$ Eq (4)

where *n* is number of runner blades and *N* is runner speed in RPM. Assuming a fish will be struck by a blade if not passing through the imaginary cylinder within the $t_{cr}$, the probability of blade strike is estimated as:

$P= \frac{t}{t_{cr}}= \frac{l\cdot sin\theta\cdot n\cdot(\frac{N}{60})}{V_{r}}$ Eq (5)
